# Federal and state cooperation necessary but not sufficient for effective regional mental health systems: insights from systems modelling and simulation

**DOI:** 10.1038/s41598-021-90762-x

**Published:** 2021-05-27

**Authors:** Jo-An Occhipinti, Adam Skinner, Samantha Carter, Jacinta Heath, Kenny Lawson, Katherine McGill, Rod McClure, Ian B. Hickie

**Affiliations:** 1 Systems Modelling, Simulation, and Data Science, Brain and Mind Centre, Faculty of Medicine and Health, University of Sydney, 94 Mallet Street, Camperdown, NSW Australia; 2 Computer Simulation & Advanced Research Technologies (CSART), Sydney, Australia; 3 Menzies Centre for Health Policy, University of Sydney, Sydney, Australia; 4 Translational Health Research Institute, Western Sydney University, Penrith, Australia; 5 Hunter New England & Central Coast Primary Health Network, Newcastle, Australia; 6 Everymind, Hunter New England Local Health District, Newcastle, Australia; 7 Centre for Brain and Mental Health Research, University of Newcastle, Callaghan, Australia; 8 MH-READ, Hunter New England Local Health District, Newcastle, Australia; 9 Faculty of Medicine and Health, University of New England, Armidale, Australia

**Keywords:** Disease prevention, Health policy, Health services, Public health

## Abstract

For more than a decade, suicide rates in Australia have shown no improvement despite significant investment in reforms to support regionally driven initiatives. Further recommended reforms by the Productivity Commission call for Federal and State and Territory Government funding for mental health to be pooled and new Regional Commissioning Authorities established to take responsibility for efficient and effective allocation of ‘taxpayer money.’ This study explores the sufficiency of this recommendation in preventing ongoing policy resistance. A system dynamics model of pathways between psychological distress, the mental health care system, suicidal behaviour and their drivers was developed, tested, and validated for a large, geographically diverse region of New South Wales; the Hunter New England and Central Coast Primary Health Network (PHN). Multi-objective optimisation was used to explore potential discordance in the best-performing programs and initiatives (simulated from 2021 to 2031) across mental health outcomes between the two state-governed Local Health Districts (LHDs) and the federally governed PHN. Impacts on suicide deaths, mental health-related emergency department presentations, and service disengagement were explored. A combination of family psychoeducation, post-attempt aftercare, and safety planning, and social connectedness programs minimises the number of suicides across the PHN and in the Hunter New England LHD (13.5% reduction; 95% interval, 12.3–14.9%), and performs well in the Central Coast LHD (14.8% reduction, 13.5–16.3%), suggesting that aligned strategic decision making between the PHN and LHDs would deliver substantial impacts on suicide. Results also highlighted a marked trade-off between minimising suicide deaths versus minimising service disengagement. This is explained in part by the additional demand placed on services of intensive suicide prevention programs leading to increases in service disengagement as wait times for specialist community based mental health services and dissatisfaction with quality of care increases. Competing priorities between the PHN and LHDs (each seeking to optimise the different outcomes they are responsible for) can undermine the optimal impact of investments for suicide prevention. Systems modelling provides essential regional decision analysis infrastructure to facilitate coordinated federal and state investments for optimal impacts.

## Introduction

For more than a decade, the rates of mental health-related emergency department (ED) presentations in Australia have been increasing across all age groups^1^, prevalence of mental disorders has remained consistent^2^, and overall suicide rates have increased from 10.4 per 100,000 in 2004 to 12.7 in 2017^3,4^. This has occurred despite a national commitment to improving mental health outcomes, decades of investments, national mental health system reforms, and successive ‘strategic’ mental health and suicide prevention action plans premised on complementing state and territory government actions and promising to deliver timely, coordinated, quality mental health care^5,6^. A range of explanations for this policy resistance have been proffered including Australia’s federated arrangements with regards to financial and service-related responsibilities for mental health care that have challenged attempts at accountability^7^, and the complexity of the mental health service system that is fragmented, difficult to navigate, and lacking coordination^8,9^.

Much has been written regarding the vital role of systematic and timely monitoring of mental health and suicide outcomes (beyond the existing service activity monitoring and the capturing of qualitative experiences of people within the health system) as a key driver of accountability, improved system performance, and continuous system-wide quality improvement in mental health care and suicide prevention^10,11^. While a robust, outcomes-focussed monitoring infrastructure is certainly needed, alone it is unlikely to deliver impact; two additional challenges will need to be overcome. Firstly, investment decisions to improve mental health outcomes and prevent suicide cannot simply rely on data monitoring. Effective decision making to address complex problems requires an appropriate predictive planning framework (underutilised in mental health system planning) to leverage this data, forecast future trajectories of population mental health and suicide outcomes, and simulate the likely impacts of alternative options for investments in programs, services and initiatives; enabling investments that are strategic rather than dispersed^9^. Systems modelling is uniquely able to capture population and demographic dynamics and changes over time in social and economic drivers of psychological distress; it captures feedback loops that drive the vicious and virtuous cycles that exist in the real world; it captures the fluctuating interplay between service supply versus demand, and workforce dynamics; and it captures change over time in individual intervention effects and the potentially non-additive effects of intervention combinations^8,12,13^. These characteristics of complex systems bedevil traditional analytic approaches^14^. Secondly, different actors in the system can have different priorities and agendas based on their source of financing, community needs, system pressures, competing views of local stakeholders, and political considerations.

As part of the most recent major reforms (2015), the Australian Government established 31 Primary Health Networks (PHNs) across the country to decentralise decision making and enable investments in programs, services and initiatives that better respond to local population needs, contexts and priorities^15,16^. Five years on from these reforms, persistent system failures have been attributed to ‘*a lack of clarity across the tiers of government about roles, responsibilities and funding, leading to persistent wasteful overlaps, yawning gaps in service provision, and limited accountability’*^17^ for mental health outcomes*.* To address this issue, further reforms have been recommended that call for Australian Government and State and Territory Government funding for mental health to be pooled and new Regional Commissioning Authorities be established to take responsibility for allocating these pooled resources and improve ‘*efficient and effective use of taxpayer money*’^17^.

On the surface, the implementation of overarching regional governance structures, pooling of resources, and systematic and timely monitoring of mental health and suicide outcomes represent an obvious and ideal solution to the problem of waste, inefficiencies, gaps in service systems and increasing accountability for investments made across the mental health system (from primary prevention programs, to primary, secondary and tertiary care services). However, this solution is unlikely to negate the need for integrated systems modelling and monitoring infrastructure to support strategic investment decisions and overcome policy resistance. Nor will it necessarily ensure the buy-in of stakeholders working across the system required for successful implementation and integration of new programs, services, and initiatives. Participatory approaches to the development of systems models^18^ and collective interaction with these models to quantify the trade-offs of alternative system strengthening strategies, offer promise in helping to achieve alignment of agendas for collaborative, coordinated, optimised, and sustained investments and actions.

This study aimed to answer three key questions; (1) what impact on suicide outcomes could be achieved for the region if the optimal combination of programs and initiatives were identified and implemented; (2) if the priorities of the PHN and two LHD were aligned (i.e., focussed on reducing suicide deaths), would independent decision making regarding the optimal combination of programs and initiatives for the PHN catchment as a whole or LHD sub-regions deliver similar impacts on suicide outcomes; (3) if the priorities of the PHN and two Local Health Districts (LHDs) were not aligned (i.e., each were seeking to optimise the different outcomes they are responsible for), would impact on suicide outcomes for the region be undermined. In addressing these questions, this study will inform optimal strategies for overcoming policy resistance and delivering significant reductions in suicide for the region and will inform whether the pooling of funds and/or the aligning of agendas across national- and state-governed agencies is important.

## Method

### Context

The Hunter New England and Central Coast (HNECC) PHN is the second largest PHN in New South Wales, covering 133,812 square kilometres, reaching from just north of Sydney, across the north west of NSW, to the Queensland border. It incorporates 23 Local Government Areas (LGAs) and is serviced by the Hunter New England Local Health District (HNE LHD) and Central Coast Local Health District (CC LHD). In 2018, estimated resident population for the HNECC PHN catchment was 1,269,782^19^. Within the HNECC PHN region, a range of LGAs have significant levels of relative disadvantage^20^. In 2017–2018, the rate at which people aged 18 years and over experienced high or very high psychological distress was 13.5 per 100, higher than the NSW (12.4 per 100) and Australian (12.9 per 100) averages^19^. Over the period 2013–2017 there were a total of 764 (13.7 per 100,000) deaths from suicide and self-inflicted injuries, which was higher than both NSW (10.5 per 100,000) and Australia (12.2 per 100,000) for the same time period^19^. These high rates of suicide in the region prompted the two LHDs (Hunter New England LHD and Central Coast LHD that fall within the PHN catchment) to come together with the HNECC PHN to explore how best to invest in coordinated actions to reduce suicidal behaviour and other key mental health indicators in the region. The functions and responsibilities of PHNs (formally known as Medicare Locals) and LHDs (also known as Local Health Networks) in the Australian context is well described^21,22^.

### Model structure, inputs, and outcome indicators

**Figure 1:**
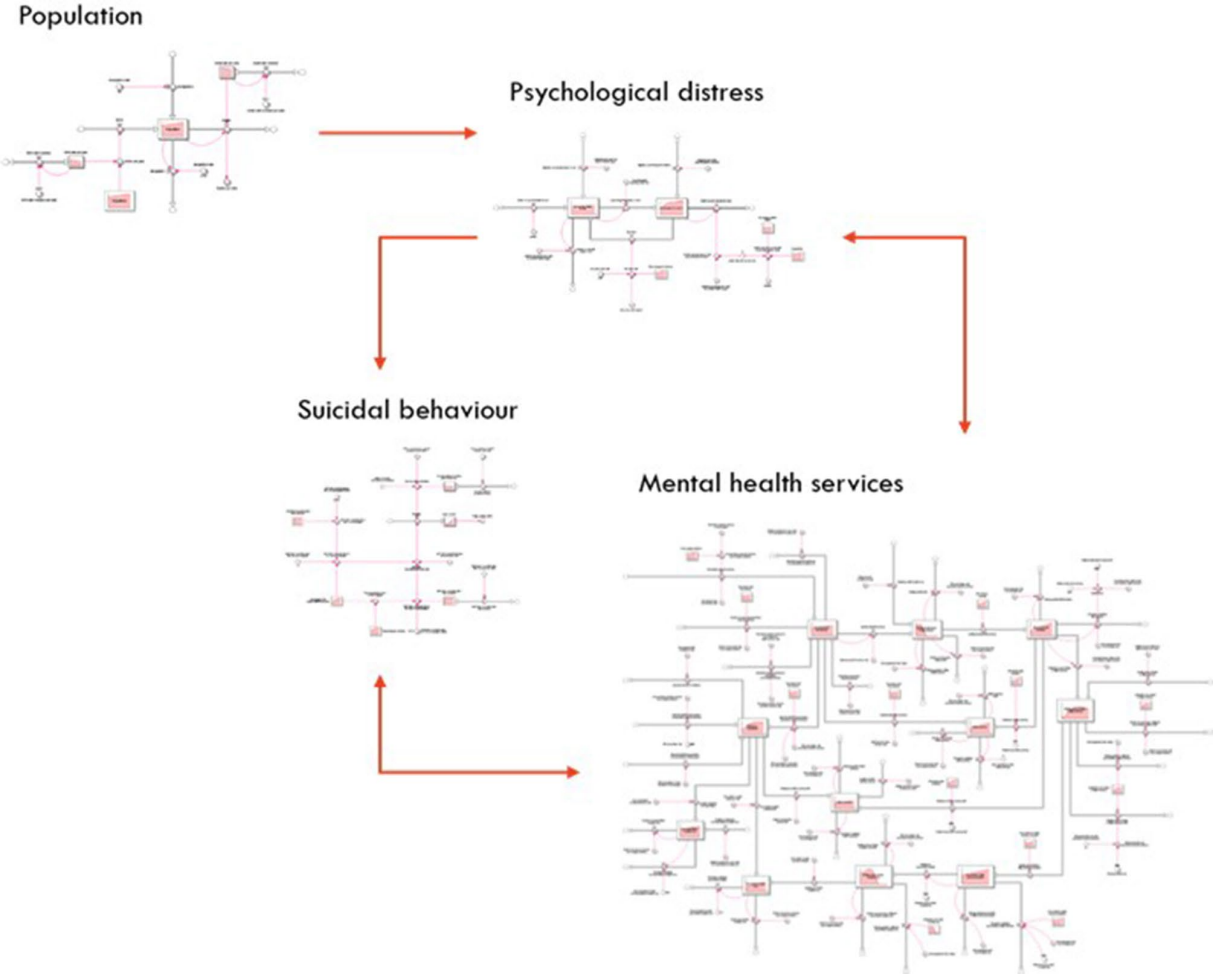
A high-level overview of the causal structure and pathways of the system dynamics model.

A system dynamics model was developed using a participatory modelling approach (described in Additional file 1, along with details of the core model structure, parameter estimates, data sources and assumptions). The core model structure was similar to a model previously described^9^ and included: (1) a population component, capturing changes over time in the size of the population resulting from births, migration, and mortality; (2) a psychological distress component that models flows of people to and from states of low or no psychological distress (Kessler 10 [K10] scores below 15), and moderate to very high psychological distress (K10 score 16–50); (3) a mental health services component that models the movement of psychologically distressed people through one of several possible service pathways across the primary to tertiary service continuum involving (potentially) general practitioners, psychiatrists and allied mental health professionals (including psychologists, mental health nurses, social workers etc.), psychiatric inpatient care, community mental health centres, and online services; and (4) a suicidal behaviour component that captures self-harm hospitalisations (used as a proxy for suicide attempts—see limitations) and suicide deaths. Figure 1 provides a high-level overview of the causal structure and pathways of the model.

Primary model outputs included total (cumulative) numbers of self-harm hospitalisations and suicide deaths, and self-harm hospitalisation and suicide rates per 100,000 population. The model also provided estimates of the prevalence of moderate to very high psychological distress and the total (cumulative) mental health-related ED presentations and psychiatric hospitalisations, and a range of measures of mental health service usage (e.g., mental health-related general practice consultations, psychiatrist or allied mental health services capacity, services waiting times, and the numbers of psychologically distressed consumers that have disengaged from treatment). All outputs were calculated every 0.4375 days (i.e., one sixteenth of a week) over a period of 28 years, starting from 1 January 2011, permitting comparisons of model outputs with historic data from 2011 to 2017 and forecasts of the impacts of intervention scenarios described below simulated over a 10-year period from 2021 to 2031.

Parameter values that could not be derived directly from available data or published research were estimated via constrained optimisation, implemented in Stella Architect ver. 1.9.4, using historical time series data on the prevalence of psychological distress, self-harm hospitalisations and suicide rates, and mental health-related service usage (subsidised general practice consultations and allied mental health services claimed per year, psychiatric bed occupancy). Powell’s method was employed to obtain the set of (optimal) parameter values minimising the sum of the mean absolute percent error calculated for each time series separately (i.e., the mean of the absolute differences between the observed time series values and the corresponding model outputs, where each difference is expressed as a percentage of the observed value).

### Modelled programs, services, and initiatives

In addition to the ability to scale up or down mental health services capacity captured in the core model structure, a range of mental health and suicide prevention programs and initiatives selected by the participating stakeholders were integrated into the model (Table 1). These programs and initiatives were identified based on current local priorities, contextual relevance, and feasibility of implementation. Details of each program, service and initiative, their default parameter estimates, and the data and evidence used to inform these estimates are provided in Additional file 1, Table S1. A model interface was developed to facilitate comparison of the impact of intervention scenarios against a baseline (business as usual) scenario, in which existing policies and programs remain in place and mental health services capacity continues to increase at current rates.

**Table 1:** Description of interventions examined (additional details regarding evidence and parameters used are provided in the Supplementary Materials).

Intervention	Description
**1. Mental health & suicide prevention interventions**	
Post-attempt assertive aftercare	Post-attempt assertive aftercare is an active outreach and enhanced contact program to reduce readmission in those presenting to services after a suicide attempt. It is implemented through existing community-based mental healthcare (CMHC) services and includes individually tailored contact, solution focused counselling, and motivations to adherence to follow-up treatments and continuity of contact
GP training	Short (1–2 days) training programs aimed at reducing suicidal ideation through referral to specialised psychiatric services. This includes people who may be thinking about suicide for the first time or have survived a previous attempt
Community-based education programs	Community-based education programs aim to improve recognition of suicide risk and increase help seeking through improved understanding of the causes and risk factors for suicidal behaviour. The effectiveness of this intervention is assumed to increase with increasing community support due to greater opportunity for identification of at-risk individuals by community and organisational gatekeepers
Family education and support	Provision of education and support to families and carers of patients presenting to or engaged with mental health services, with the aim of supporting family or carer involvement in the management of mental disorders
Safety planning	Safety planning aims to reduce suicidal behaviour through the provision of a specific plan for staying safe during crisis to suicidal patients presenting to an emergency department. The modelled intervention also includes up to 2 follow-up phone calls to monitor suicide risk and support treatment engagement
Safe space alternative to emergency departments	Based on the United Kingdom’s Safe Haven café model, this intervention provides an alternative point of contact with mental health services for people experiencing acute psychological distress who may otherwise present to an emergency department
Social connectedness programs	Community support programs and services that increase social connectedness, reducing isolation and enhancing resilience in the face of adversity
Community-based acute care services	Responsive clinical mental health services delivered by community mental health teams. People in crisis may call and request either a home-based visit or a centre-based visit, depending on their level of functioning and risk
**2. Services capacity increases**	
GP mental health services	Multiplies the annual rate of increase in the total number of mental health-related GP consultations that can be completed per week. The default value (1) corresponds to the business as usual case, in which services capacity continues to increase at the current rate, estimated using Medicare Benefits Schedule (MBS) data for 2012–2017 assuming services were operating at (near-) maximum capacity over this period
Psychiatrists and allied services	Multiplies the annual rate of increase in the total number of psychiatrist and allied services that can be provided per week. The default value (1) corresponds to the business as usual case, in which services capacity continues to increase at the current rate, estimated using Medicare Benefits Schedule (MBS) data for 2012–2017 assuming services were operating at (near-) maximum capacity over this period
Psychiatric hospital care	Multiplies the annual rate of increase in the maximum number of psychiatric hospital admissions per week. The default value (1) corresponds to the business as usual case, in which services capacity continues to increase at the current rate, estimated using hospital separations data for 2011–2018 available from HealthStats NSW (http://www.healthstats.nsw.gov.au) and data on the provision of specialised psychiatric care in public hospitals published by the Australian Institute of Health and Welfare (available at: https://www.aihw.gov.au/reports-data/health-welfare-services/mental-health-services/data)
Community mental healthcare services	The annual increase in the total number of community mental health service contacts per 10,000 population that can be provided per week. The default value (0, corresponding to no capacity growth) was derived from service usage data for 2008–2017 published by the Australian Institute of Health and Welfare (available at: https://www.aihw.gov.au/reports-data/health-welfare-services/mental-health-services/data)

Potential discordance in the best-performing intervention scenarios across mental health outcomes and between the two LHDs was assessed by examining reductions in total (cumulative) numbers of suicides, mental health-related ED presentations, and patients disengaging from services under all (495) possible combinations of four interventions selected from the 12 programs, services and initiatives modelled. Differences in projected numbers of suicides, ED presentations, and disengagements from services between the baseline scenario and each intervention scenario across the PHN and in the two LHDs were calculated for the period 2021–2031. We identified the best-performing (optimal) combinations of interventions for each outcome across the PHN and in each LHD (i.e., combinations of interventions minimising the total number of suicides, ED presentations, or patients disengaging), as well as all intervention combinations performing better than every other combination on at least one outcome; these latter combinations, corresponding to non-dominated or Pareto optimal solutions, may be considered as equally optimal alternatives where the goal is to simultaneously minimise suicide mortality, ED presentations, and disengagement from care (see, e.g., Branke et al.^23^). Analyses in which five interventions were selected from the 12 interventions modelled yielded results similar to those for combinations of four interventions, indicating that our general conclusions are not dependent upon the choice of intervention set size (see “Results” section).

Sensitivity analyses were performed to assess the impact of uncertainty in estimates of the direct effects of each intervention and the duration of increased psychological distress incidence due to coronavirus-related unemployment and social dislocation on the simulation results. We used Latin hypercube sampling to draw 100 sets of values for selected model parameters determining the direct effects of the interventions and the duration of the COVID-19 pandemic effects from a relatively broad distribution of values (± 20% of the default values for the direct intervention effects, ± 50% of the default value for the duration of the pandemic effects). Differences in projected numbers of suicides, ED presentations, and patients disengaging from services between the baseline and intervention scenarios were calculated for each set of parameter values and summarised using simple descriptive statistics.

### Ethics approval and consent to participate

This modelling study did not require ethics approval.

### Consent for publication

Not applicable.

## Results

**Figure 2:**
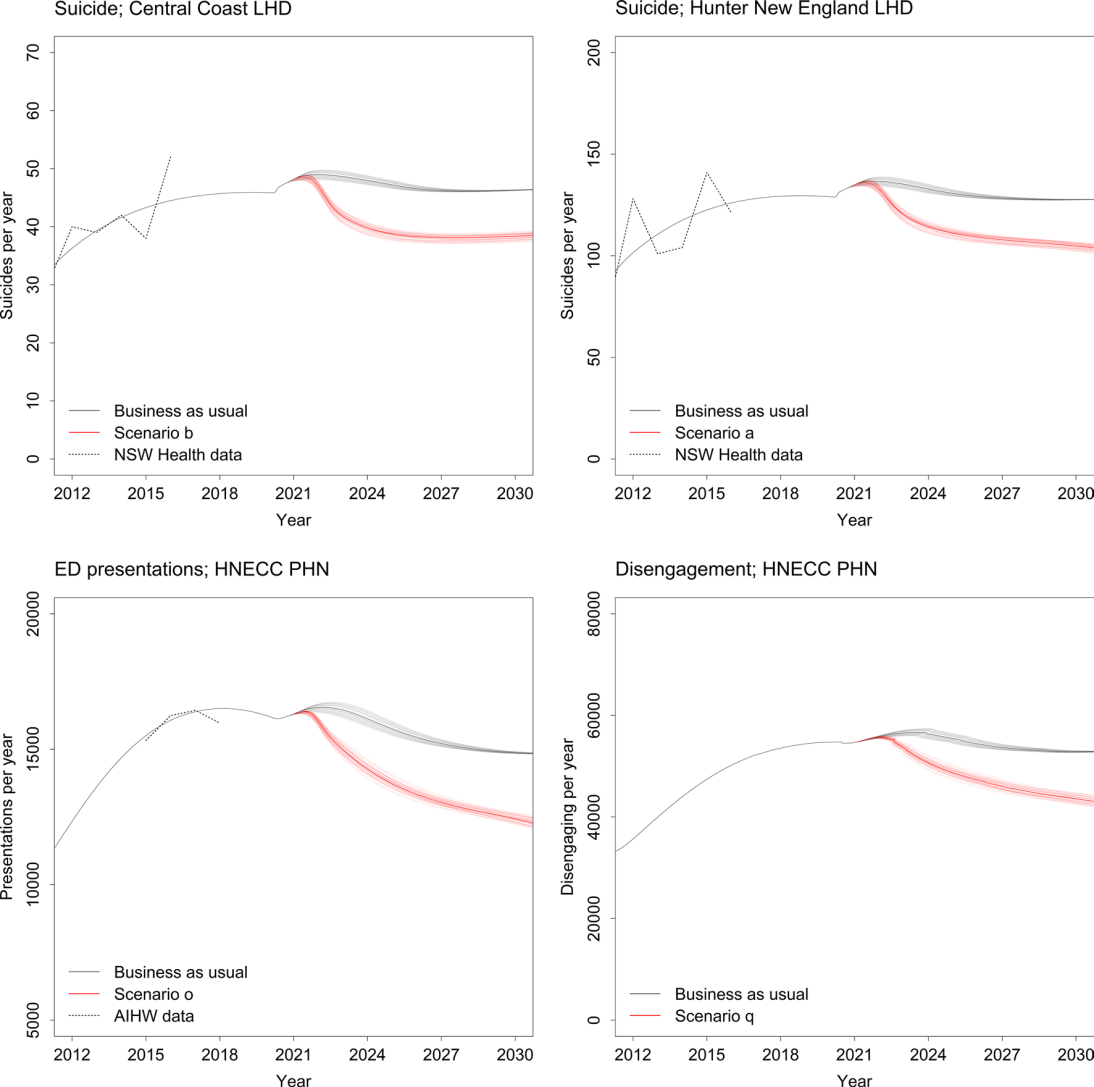
Numbers of suicides, emergency department (ED) presentations, and patients disengaging from services per year under the baseline scenario (i.e., business as usual) and the optimal intervention scenario for each outcome (see Table 2 for intervention scenario details). The dotted lines show estimates of numbers of suicides and ED presentations published by NSW Health (http://www.healthstats.nsw.gov.au) and the Australian Institute of Health and Welfare (2019). Model outputs from the sensitivity analyses, incorporating uncertainty in the intervention effects and the duration of increased distress onset due to the COVID-19 pandemic, are shown as lighter solid lines; the heavier solid lines show model outputs obtained assuming the default parameter values.

Projected numbers of suicides, mental health-related ED presentations, and patients disengaging from services per year under the baseline (business as usual) scenario are presented in Fig. 2. Numbers of suicides for the HNECC PHN are projected to increase from 174.9 suicides per year in early 2020 (prior to the effects of coronavirus-related lockdowns on unemployment and psychological distress commencing in March 2020) to 186 suicides per year in late 2021, before declining to 174 suicides per year in 2031. Mental health-related ED presentations across the PHN are projected to increase from 16,210 presentations per year at the start of 2020 to a maximum of 16,552 presentations per year in early 2022 and then decrease to 14,841 presentations per year in 2031. Patient disengagement from services follows a similar pattern, increasing from nearly 55,000 disengagements per year (for the PHN as a whole) at the beginning of 2020 to 56,668 disengagements per year in early 2024, before decreasing to pre-COVID values at the end of the forecast period (1 January 2031).

**Figure 3:**
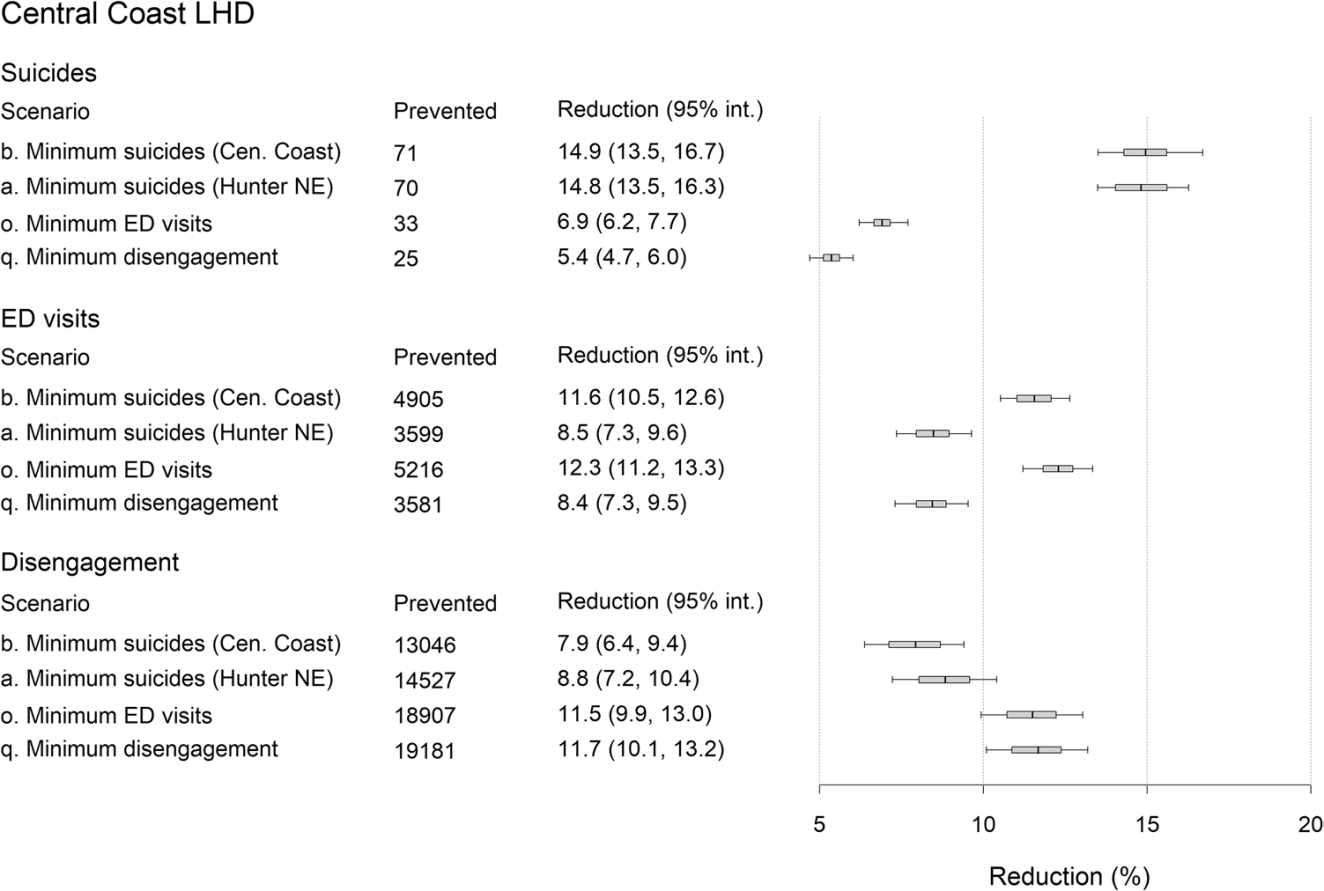
Projected reductions (%, relative to business as usual) in total numbers of suicide deaths, mental health-related emergency department (ED) presentations, and patients disengaging from services in the Central Coast Local Health District (LHD) over the period 2021–2031. Results are shown for the optimal intervention scenario(s) for each outcome (see Table 2 for intervention scenario details). Mean percentage reductions and 95% intervals reported in the rightmost column were derived from the distributions of projected outcomes calculated in the sensitivity analyses (note that the 95% intervals provide a measure of the impact of uncertainty in the assumed intervention and pandemic effects, but should not be interpreted as confidence intervals). Numbers of cases (i.e., suicides, ED presentations, and disengagements from services) prevented were obtained assuming the default parameter values. Mean percentage reductions and 50% and 95% intervals are plotted on the right.

**Figure 4:**
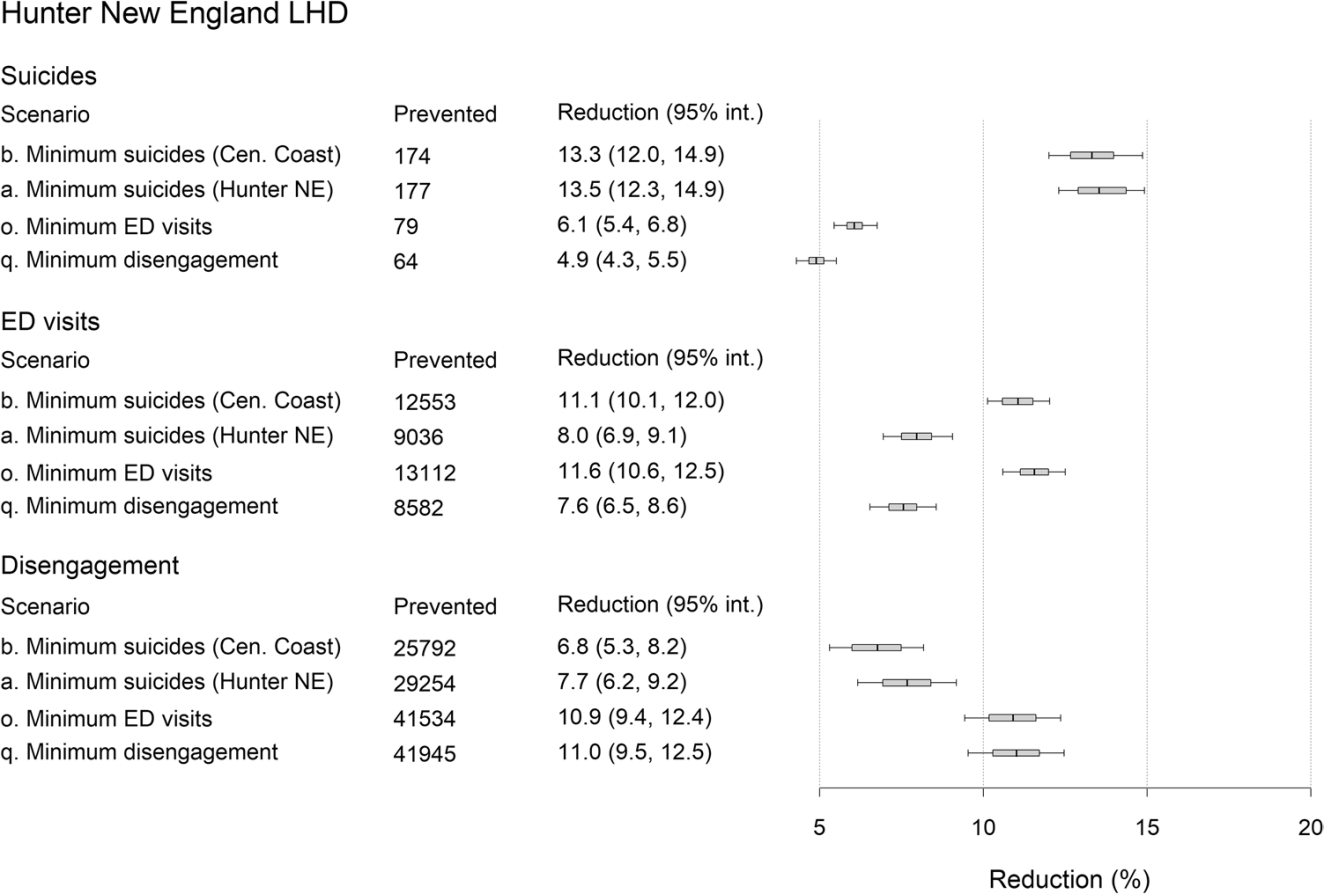
Projected reductions (%, relative to business as usual) in total numbers of suicides, mental health-related emergency department (ED) presentations, and patients disengaging from services in the Hunter New England Local Health District (LHD) over the period 2021–2031. Results are shown for the optimal intervention scenario(s) for each outcome (see Table 2 for intervention scenario details). Mean percentage reductions and 95% intervals reported in the rightmost column were derived from the distributions of projected outcomes calculated in the sensitivity analyses (note that the 95% intervals provide a measure of the impact of uncertainty in the assumed intervention and pandemic effects, but should not be interpreted as confidence intervals). Numbers of cases (i.e., suicides, ED presentations, and disengagements from services) prevented were obtained assuming the default parameter values. Mean percentage reductions and 50% and 95% intervals are plotted on the right.

**Figure 5:**
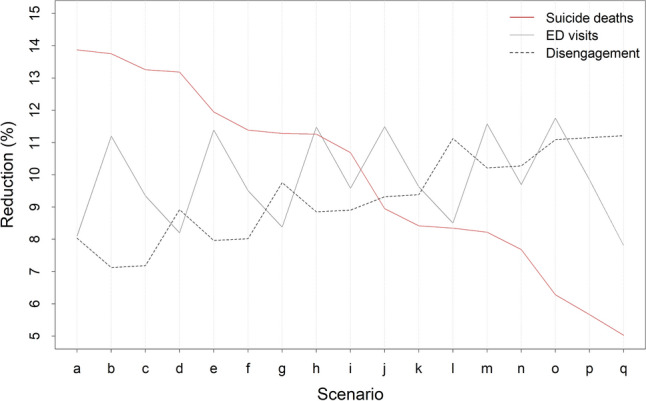
Projected reductions (%, relative to the baseline scenario) in total numbers of suicides, mental health-related emergency department (ED) presentations, and patients disengaging from services across the Hunter New England and Central Coast Primary Health Network over the period 2021–2031. Intervention scenarios are ordered so that the number of suicides prevented decreases from left to right (see Table 2 for intervention scenario details).

Under the business-as-usual scenario, 1778 suicides, 155,901 mental health-related ED presentations, and 544,972 disengagements from services are projected for the PHN over the forecast period (i.e., 1 January 2021 to the beginning of 2031; see Table 2), including 471 suicides, 42,423 ED presentations, and 164,293 disengagements from services in the Central Coast LHD, and 1307 suicides, 113,478 ED presentations, and 380,678 disengagements from services in the Hunter New England LHD. A combination of community-based acute care services, family psychoeducation, post-attempt care, and safety planning (intervention scenario b) minimises the number of suicides in the Central Coast LHD, while a combination of family psychoeducation, post-attempt care, safety planning, and social connectedness programs (intervention scenario a) prevents the greatest number of suicides across the PHN and in the Hunter New England LHD (see Figs. 2, 3, 4 and 5). Projected numbers of ED presentations and patients disengaging from services are minimised (in both LHDs and the PHN as a whole) under intervention scenarios combining family psychoeducation, social connectedness programs, an increase in community mental health care services capacity, and either community-based acute care services (intervention scenario o, minimising numbers of ED presentations) or an increase in general practitioner (GP) services capacity (intervention scenario q, minimising disengagement) (see Figs. 2, 3, 4 and 5).

**Table 2:** Non-dominated solutions for the hunter New England and central coast primary health network (HNECC PHN).

Intervention scenario		Suicides (% reduction)		ED presentations (% reduction)		Disengagements (% reduction)	
0	Business as usual (no interventions)	1778		155,901		544,972	
a	Family psychoeducation, post-attempt care, safety planning, social connectedness	1532	(13.8)	143,266	(8.1)	501,191	(8.0)
b	Acute care services, family psychoeducation, post-attempt care, safety planning	1534	(13.7)	138,444	(11.2)	506,134	(7.1)
c	Family psychoeducation, post-attempt care, safety planning, safe space services	1543	(13.2)	141,327	(9.3)	505,835	(7.2)
d	Family psychoeducation, post-attempt care, safety planning, CMHC services capacity increase	1544	(13.2)	143,114	(8.2)	496,362	(8.9)
e	Acute care services, family psychoeducation, post-attempt care, social connectedness	1566	(11.9)	138,145	(11.4)	501,555	(8.0)
f	Family psychoeducation, post-attempt care, safe space services, social connectedness	1576	(11.4)	141,079	(9.5)	501,267	(8.0)
g	Family psychoeducation, post-attempt care, social connectedness, CMHC services capacity increase	1578	(11.2)	142,842	(8.4)	491,787	(9.8)
h	Acute care services, family psychoeducation, post-attempt care, CMHC services capacity increase	1578	(11.2)	138,001	(11.5)	496,730	(8.9)
i	Family psychoeducation, post-attempt care, safe space services, CMHC services capacity increase	1588	(10.7)	140,963	(9.6)	496,443	(8.9)
j	Acute care services, family psychoeducation, safety planning, social connectedness	1619	(8.9)	137,991	(11.5)	494,169	(9.3)
k	Family psychoeducation, safety planning, safe space services, social connectedness	1629	(8.4)	140,891	(9.6)	493,829	(9.4)
l	Family psychoeducation, safety planning, social connectedness, CMHC services capacity increase	1630	(8.3)	142,641	(8.5)	484,337	(11.1)
m	Acute care services, family psychoeducation, safety planning, CMHC services capacity	1632	(8.2)	137,852	(11.6)	489,305	(10.2)
n	Family psychoeducation, safety planning, safe space services, CMHC services capacity increase	1642	(7.6)	140,782	(9.7)	488,964	(10.3)
o	Acute care services, family psychoeducation, social connectedness, CMHC services capacity increase	1667	(6.2)	137,573	(11.8)	484,531	(11.1)
p	Family psychoeducation, safe space services, social connectedness, CMHC services capacity increase	1678	(5.6)	140,555	(9.8)	484,195	(11.2)
q	Family psychoeducation, social connectedness, GP services capacity increase, CMHC services capacity increase	1689	(5.0)	143,738	(7.8)	483,846	(11.2)

Each solution is a combination of four interventions that performs better than all other combinations on at least one outcome, preventing more suicides, mental health-related emergency department (ED) presentations, and/or disengagement. Numbers of suicides, mental health-related ED presentations, and patients disengaging across the PHN over the period 2021–2031 are presented for each intervention scenario.

Figures 3 and 4 present percentage reductions in numbers of suicides, mental health-related ED presentations, and patients disengaging (compared to business as usual) under the optimal intervention scenarios for each outcome. The intervention combinations minimising numbers of suicides in the Central Coast LHD and Hunter New England LHD (intervention scenarios b and a, respectively) yield very similar reductions in suicide mortality in each LHD, preventing 13.3–14.9% of suicides projected under the baseline scenario over the period 2021–2031. Percentage reductions in suicide mortality under the intervention scenarios minimising numbers of ED presentations (scenario o) and disengagement from services (scenario q) are substantially lower, ranging from 4.9% to 6.9%. The combinations of interventions that are most effective in preventing suicides in the Central Coast LHD and mental health-related ED presentations across the PHN produce similar reductions in numbers of ED visits in each LHD (11.1–11.6% for scenario b, 11.6–12.3% for scenario o), while the intervention combinations minimising suicide mortality in the Hunter New England LHD and disengagement in both LHDs prevent significantly fewer presentations to emergency departments (7.6–8.5%). Reductions in patient disengagement achieved under the best-performing intervention combination (i.e., scenario q, 11.0–11.7%) are similar to those projected under the intervention combination minimising numbers of ED presentations (10.9–11.5%) but are considerably greater than reductions in patient disengagement projected under the optimal intervention combinations for suicide prevention (6.8–8.8%).

Among the 495 possible combinations of four interventions selected from the 12 interventions modelled, 17 intervention combinations may be considered optimal in that they perform better than all other intervention combinations in preventing suicides, mental health-related ED presentations, and/or disengagement from services across the PHN (see Table 2, Figure S17). Reductions in projected numbers of suicides, ED presentations, and patients disengaging from services for each of these non-dominated solutions are presented in Fig. 5. Combinations of interventions that are more effective in preventing suicides (those towards the left of Fig. 5) generally perform poorly (i.e., relative to other solutions) in reducing disengagement, and vice versa. Relationships between reductions in projected numbers of ED presentations and both suicide mortality and disengagement are more haphazard, showing no clearly identifiable pattern. Analyses in which five interventions are selected from the 12 modelled interventions yield results qualitatively similar to those in Fig. 5 (see Fig. S18).

## Discussion

This study harnessed systems modelling and simulation to explore whether the recent Productivity Commission recommendations to pool resources under a unifying regional governance structure would be sufficient to prevent persistent policy resistance. Findings offer promise that policy resistance can be overcome with implementation of the optimal mix of programs and initiatives and indicate that reductions in suicide deaths in the order of 14.2–16% over 10 years are achievable across the region, however, the scale of this potential impact falls well short of the ambitious Premier’s Target to reduce the rate of suicide deaths in NSW by 20% by 2023^24^, as the first step in the journey towards zero suicides. Results also revealed that that while the best performing combination of interventions to minimise suicide deaths differed between the two LHDs, the difference in the number of suicides prevented was negligible, suggesting that optimal impact of interventions could be achieved through independent strategic decision making at regional (PHN) or sub-regional (LHD) levels if agendas are aligned in prioritising suicide prevention. In contrast, competing priorities between the PHN and LHDs (such as minimising mental health-related ED presentations or service disengagement) can undermine optimal impacts of investments to prevent suicide deaths. Understanding such trade-offs and their implications for mental health outcomes and suicide prevention is not possible without the ability to forecast comparative impacts of alternative investment strategies. Therefore, in short, the Productivity Commission’s recently recommended pooling of national and state funding for mental health under new Regional Commissioning Authorities is neither necessary nor sufficient to prevent ongoing policy resistance.

A unifying regional governance structure will not negate the need for advanced decision analytic capability provided by systems modelling and simulation to identify optimal combinations of programs and initiatives for suicide prevention, as well as consensus building processes to unite regional stakeholders behind an agreed agenda. Studies have highlighted the importance of political support from local authorities and acceptance of proposed interventions by the local community for successful implementation of public health programs^25,26^. Securing the required leadership and sustained support for effective, coordinated implementation of best performing interventions identified by systems modelling requires representatives from across the regional mental health and social systems and broader community to come together to debate, weigh up the trade-offs, and prioritise the key mental health outcomes to be addressed. Regardless of whether proposed reforms are implemented in Australia, the participatory systems modelling approach will be essential for coordinating regional investments in mental health system strengthening and suicide prevention and reducing fragmentation. The participatory systems modelling approach would support the integrated decision analytic, monitoring and evaluation ecosystem needed to achieve the transparency and accountability required for more effective mental health outcomes both regionally and nationally.

Finally, the unintuitive finding of a marked trade-off between minimising suicide deaths versus minimising service disengagement (i.e., both cannot be optimised simultaneously) is explained in part by the additional demand placed on the regional mental health service systems of intensive suicide prevention programs. For example, post suicide attempt assertive aftercare is an active outreach and enhanced contact program designed to intensively support those presenting to services after a suicide attempt in order to prevent re-attempt. It is implemented through existing community-based mental healthcare (CMHC) services. In the context of restrictions in responsive expansion of regional workforces, new programs introduced consume existing service capacity. This in turn leads to increases in service disengagement as wait times for specialist community based mental health services and dissatisfaction with quality of care increases.

### Limitations

There are several limitations that require consideration when interpreting the findings of this study. There is potential measurement bias in the range of secondary data used to parameterise the model including the population health surveys, Medicare claims data, and PHN and Local Health District (LHD) datasets. The model acknowledges these potential sources of measurement bias and a number of commonly used strategies were employed to address them, including the triangulation of multiple data sources, parameter estimation via constrained optimisation, and local verification to identify plausible estimates.

In addition, there is potentially an under-enumeration of suicide cases used to calibrate the model, due to the misclassification of suicides to ICD codes relating to unintentional injury and events of ‘undetermined intent.’ Also, in the absence of a direct measure of suicide attempts, self-harm hospitalisations are used as a proxy in this study. Suicide attempts identified from hospital admissions data likely only capture those cases serious enough to warrant medical intervention, and instances of self-harm where the intent wasn’t clear may be not coded as suicide attempts. However, this under-enumeration is consistent across simulations of the baseline case and intervention scenarios and as such is unlikely to affect the forecast estimates of impact (i.e., the % reduction in suicidal behaviour) of intervention strategies or the strategic insights derived from the model. Ongoing systematic monitoring and evaluation can determine the extent to which the model forecasts are corresponding with real-world outcomes over time, allowing refinement of model parameters to improve forecasting capabilities. Finally, as the impacts of simulated scenarios are subject to the population, demographic, behavioural, and service dynamics of the modelled region, they may not be generalisable to other regions.

### Strengths

The suite of programs, services and initiatives used in this analysis by no means represents an exhaustive list of potentially effective strategies, nor are these optimisation results intended to provide ‘the answer.’ Rather, this work demonstrates how systems modelling can provide a quantitative framework for bringing together a body of evidence, data, and local knowledge in a way that answers questions that cannot be achieved through analysis of any single dataset or through real world experimentation. A further strength of the approach is the ability to bring together representatives from different parts of the mental health system, funded by different levels of government, and engage them in a process that helps them better understand the upstream and downstream implications of investments designed to strengthen a particular part of the system. Strategy dialogues supported by a robust, objective systems modelling platform promotes communication and relationship building between system actors that traditionally do not interact. This approach also promotes the aligning of agendas needed for collaborative and coordinated action. Systems models can be iteratively refined and informed by (and will in turn inform) data collected from relevant surveys, administrative data sets, and new research. Following initial model development, key indicators for ongoing monitoring are identified, often engaging different jurisdictions and diverse stakeholders (including lived experience groups) in the process of strengthening their regional decision support asset. This not only improves the predictive capabilities of systems models over time but has the potential to keep system actors invested in sustaining their engagement with the model as a long term, collective, regionally-based decision support asset, and as an objective, constructive platform for supporting ongoing cooperation.

## Conclusion

Decades of investments, statutory inquiries, mental health system reforms, and strategic action plans, have failed to reduce suicide rates in Australia. This study has demonstrated that competing priorities between PHNs and LHDs can undermine the optimal impact of investments for suicide prevention and that the Productivity Commission’s recently recommended pooling of national and state funding for mental health under new Regional Commissioning Authorities is neither necessary nor sufficient to prevent ongoing policy resistance. Systems modelling provides essential regional decision analysis infrastructure to facilitate optimally coordinated federal and state investments.

## Supplementary Information

Supplementary Information.

## Data Availability

The datasets generated and analysed during the current study are available from the corresponding author on reasonable request.
